# Development of an Indicator to Monitor Mediterranean Wetlands

**DOI:** 10.1371/journal.pone.0122694

**Published:** 2015-03-31

**Authors:** Antonio Sanchez, Dania Abdul Malak, Anis Guelmami, Christian Perennou

**Affiliations:** 1 European Topic Centre, University of Málaga, Málaga, Spain; 2 Tour du Valat Research Centre for the Conservation of Mediterranean Wetlands, Arles, France; Shandong University, CHINA

## Abstract

Wetlands are sensitive ecosystems that are increasingly subjected to threats from anthropogenic factors. In the last decades, coastal Mediterranean wetlands have been suffering considerable pressures from land use change, intensification of urban growth, increasing tourism infrastructure and intensification of agricultural practices. Remote sensing (RS) and Geographic Information Systems (GIS) techniques are efficient tools that can support monitoring Mediterranean coastal wetlands on large scales and over long periods of time. The study aims at developing a wetland indicator to support monitoring Mediterranean coastal wetlands using these techniques. The indicator makes use of multi-temporal Landsat images, land use reference layers, a 50m numerical model of the territory (NMT) and Corine Land Cover (CLC) for the identification and mapping of wetlands. The approach combines supervised image classification techniques making use of vegetation indices and decision tree analysis to identify the surface covered by wetlands at a given date. A validation process is put in place to compare outcomes with existing local wetland inventories to check the results reliability. The indicator´s results demonstrate an improvement in the level of precision of change detection methods achieved by traditional tools providing reliability up to 95% in main wetland areas. The results confirm that the use of RS techniques improves the precision of wetland detection compared to the use of CLC for wetland monitoring and stress the strong relation between the level of wetland detection and the nature of the wetland areas and the monitoring scale considered.

## Introduction

In the context of freshwater management, resource mapping and inventorying are key to identify the location, biological productivity, potential multiple uses and biodiversity profiles of wetland ecosystems [1]. Traditional methods for mapping and inventorying resources at regional or national scales, mainly through fieldwork, are expensive and time-consuming [2] and frequently suffer spatial incompleteness, scalar inconsistencies and temporal uncertainties [3] [4]. The Ramsar convention highlights the importance of filling the gaps in baseline inventory, and stresses the need of developing techniques that can fill these gaps by using new technologies namely RS and GIS applications [3] [5].

Over large spatial and temporal scales, these powerful techniques are used as cost effective tools to improve knowledge on the types and conditions of wetlands [5] [6] in the context of standardizing wetland monitoring mechanisms, and for managing extensive wetlands in the context of the Ramsar Convention [7].

Accurate spatial information proved to assess efficiently natural and anthropogenic wetlands [8] and temporal imagery proved to be effective in analyzing wetland dynamics in space and time [9], making satellite imagery and RS techniques valid tools to be used by wetland managers and scientific researchers for monitoring and analyzing changes in wetlands.

In this context, this research supported the RhoMeo program (Rhone Mediterranean Observatory, http://rhomeo.espaces-naturels.fr), led by the Rhone Basin Water Authority in south-east France, in the development and testing of methods to improve the analysis of wetlands using RS techniques.

It is to note that, though very useful, RS techniques have some limitations as well. For some purposes, such as mapping and inventorying, RS can serve as a foundation or core technology, but in others, such as monitoring, hydrological modeling or generation of historical time series information, its use is normally limited to a support technology to complement ground-based programs [7].

The goal of this study is to develop an indicator to detect the extension of Mediterranean wetlands at local level based on RS techniques. In addition, the use of the indicator can support detecting changes in wetlands to improve their monitoring and assessment potentials at local scale.

## Study Area

**Fig 1:**
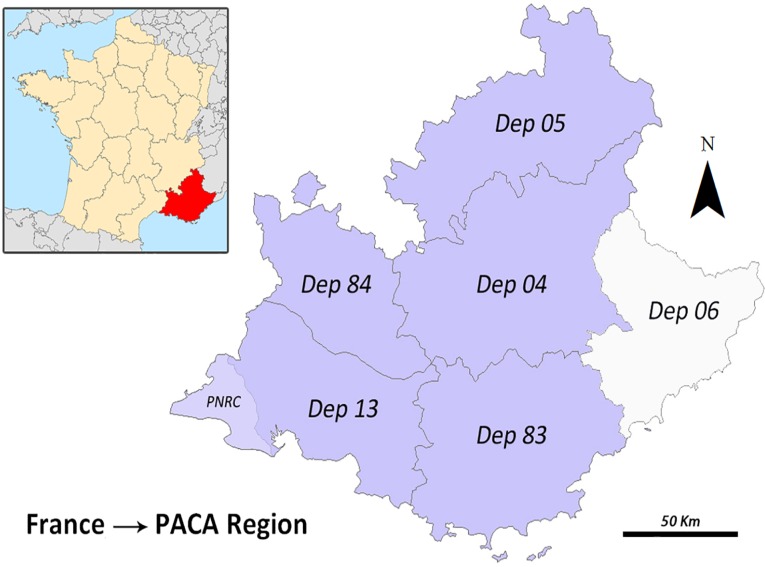
Study area. PACA region: location and departments.

The spatial framework of the study is the region Provence-Alpes-Côte d'Azur (PACA region), in southeastern France, including five of its six departments due to the availability of data (Fig 1): Dep. 04 (Alpes-de-Haute-Provence), 05 (Hautes-Alpes), 13 (Bouches-du-Rhône), 83 (Var) and 84 (Vaucluse).

The PACA region has a total surface of around 3 million ha of which around 156,000 ha are inventoried as wetlands. Around 67% of the wetland surface is located in Dep. 13 (Table 1) dominated by large wetland areas having a mean surface of 1185 ha. The Camargue Natural Regional Park (PNRC) is a wetland of international importance (under Ramsar), and is located in Department 13. The PNRC covers 82,000 ha of which 52,000 ha are wetlands. Rice fields in the PACA region are limited to this department covering a surface ranging between 10,000–15,000 ha, depending on the study year. They are included under the category “wetlands”, following the Ramsar definition used throughout this study.

**Table 1:** Percentage of total wetlands in the PACA region by department.

Dep 05	Dep 04	Dep 84	Dep 13	Dep 83	Dep 6
10.8%	13.2%	5.3%	66.8%	3.9%	-

Currently there is not data on the surface of wetlands for Dep. 6.

The size of wetlands and water bodies is highly variable, ranging between 0.05 and 12,500 ha according to the inventories. The wetlands are diverse, especially in the PNRC area where there is a mixture of natural and semi-natural wetlands: large brackish lagoons, marshes, ponds, salinas, rushes, reeds, salt meadows, rice fields, etc. Due to the region´s topography, different types exist in the study area where wetlands of the northern (Alpine) departments are composed mostly of rivers and their annexes (wet meadows, riparian woodlands) which together with lakes and dams account for a large part of the wetland surface, e.g. in departments 04 and 05, being about 24% of wetlands in the PACA.

**Fig 2:**
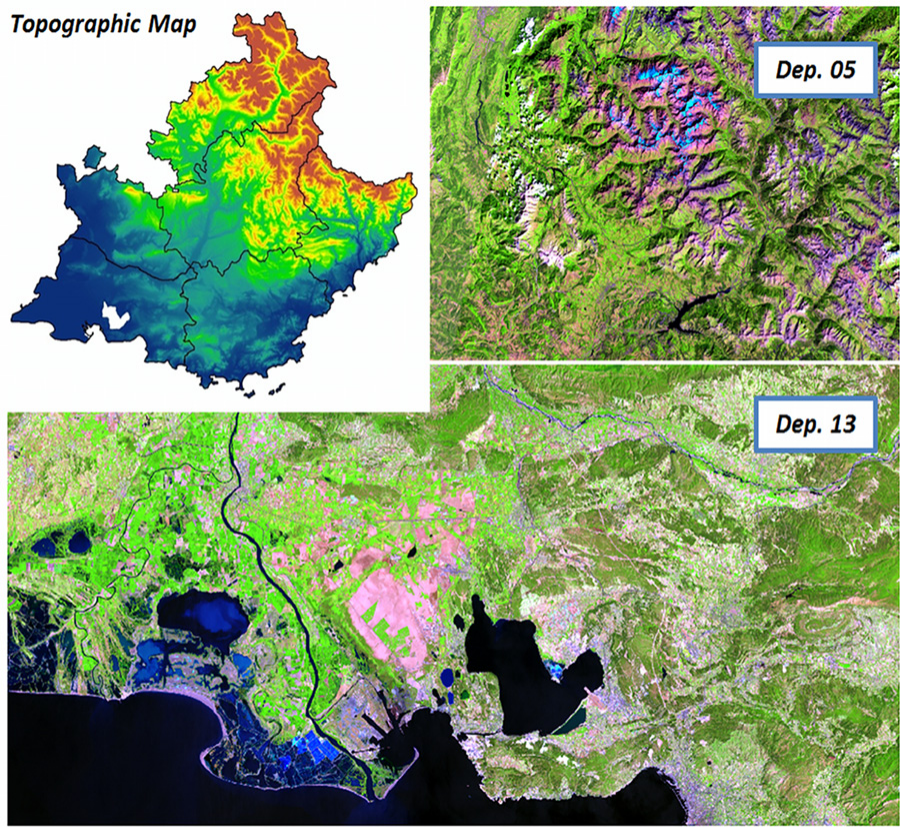
Topographic map of the PACA region and samples of satellite images (false color) of Dep. 13 (littoral) and Dep. 05 (mountainous).

Another aspect is the difference in the distribution and shape of wetlands throughout the region (Fig 2). While in the southern, coastal areas the wetlands are large and well defined, the alpine areas show a very homogeneous appearance, with wetlands mostly poorly defined or with small size and linear features, that hinders their detection by RS techniques.

## Material and Methods

### Datasets and spatial information

#### Satellite imagery

The images used in this study were Landsat satellite series (NASA, USGS) having the longest temporal record of space-based earth observations [10] and having their historical data archives freely available for research purposes. The images used have a resolution of 30 m and cover the year 1984 (Landsat 5 TM), and 2001 (7 ETM+ imagery). Between 4 and 6 images were collected for each department covering different months of both years (Table 2). We attempted to collect images on similar dates (months), paying special attention to the hydrologic cycle and the seasonality of the wetlands in the PACA region, in order to cover all seasons of the year and the different flooding stages of wetlands in the region. Due to the high presence of clouds in the images of the spring of 1984, we chose pictures of 1985.

**Table 2:** Summary of dates and properties of the satellite imagery used in the study.

Period	Satellite	Number of Bands	Bands used	Resolution	Image Date
1984	Landsat 5 TM	7 Bands	1 to 5, and 7	30 m	Jul—Aug 1984 Sep—Oct 1984 Nov—Dec 1984 Mar 1985
2001	Landsat 7 ETM+	7 Bands + 1 Panchromatic	1 to 5, and 7	30 m	Jan—Mar 2001 Apr—May 2001 Jul—Aug 2001 Oct 2001

According to the department, dates may change due to the presence of clouds in the images.

Tasseled Cap (TC) transformation [12] was applied to the Landsat imagery. The TC is inspired by the method of principal component analysis combined with a generalization from empirical observations. It transforms the Landsat bands in six principal components: the first three represent important information of the image while the others provide residual information. The TC variables used in this study were the Greenness (G) and Wetness (W) indices to separate wetland areas and water bodies from the rest of the areas. These variables have values between -1 as minimum and 1 as maximum. G is correlated with the vegetation vigor (vegetated areas are greater than zero) and W related to vegetation and soil moisture (wet areas are greater than zero).

#### Spatial data

Four layers of land reference data available for the PACA region were used for this study: the wetland inventories of the PACA Region (Fig 2), the land use/land cover (LULC) layers of the PNRC, the CLC maps of the region, and a numerical model of the territory (NMT).

**Fig 3:**
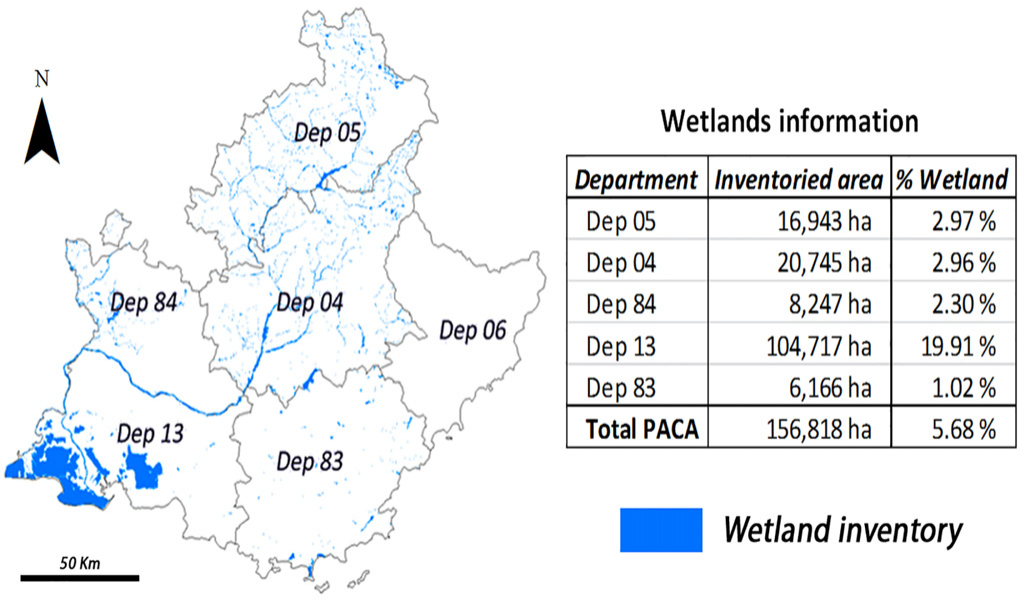
Study area: Departments of the PACA region, their relative wetland surfaces, and % covered by wetland.

The first one, the wetland inventory, displays the surface covered by water bodies and wetlands in the study area. These inventories were developed by local authorities and experts and have been validated through field-checking (except for Dep. 06, which has none). The inventories were developed between 2001 and 2012, then aggregated and they provide a reliable overview of regional wetlands for the decade 2001–2010. Inventories of Dep. 05 and 84 were not totally completed at the time of the analysis (Fig 3), and the analysis only covered their inventoried parts.

In the case of Dep. 13, a land use layer was available for the PNRC region that was used in this study, providing more comprehensiveness because the inventory for this department did not map at all the rice fields in the region. The LULC layer contains accurate and detailed information about the land use classes in the PNRC: types of agriculture, water bodies, forests, urban areas, etc. This layer was updated every 5 years by the PNRC authorities since 1991, including methodological improvements over time that however hinders diachronic comparisons. In this study we used the 2001 map.

Due to the lack of an accurate LULC layer that covers the whole PACA region, CLC layers for the years 1990 and 2000 were used whenever a deeper analysis of land uses was required. Classes under Agricultural and Urban areas, wetlands and water bodies (excluding Sea and ocean) were extracted due to the relevance to the analysis.

Monitoring the mountainous part of the PACA region presents topographical and climatic limitations due to the alpine influence in the North increasing the appearance of shadows, clouds and snow in the satellite images analyzed. This hinders the correct image analysis as it generates certain confusions in the classification process [13]. A numerical model of the territory (NMT) of 50 m resolution produced by the National Institute of Geography of France was used as ancillary data to correct these topographic effects.

### Description of the indicator

The wetland indicator aims at identifying the total surface of wetlands at a given date including wetlands, water bodies, and rice fields. In addition, it detects the wet part of the meadows category that are not categorized as such in the CLC and rather included within one meadow category without differentiation of wet and dry meadows. The analyses of this study covered the period between 1984 and 2001.

The methodology combines different classification techniques according to land coverage and uses some masking layers to exclude regions not related to the analysis. The study area was divided into three categories in order to improve the detection mechanism being water bodies, land and overlaying vegetation, and rice fields.

**Fig 4:**
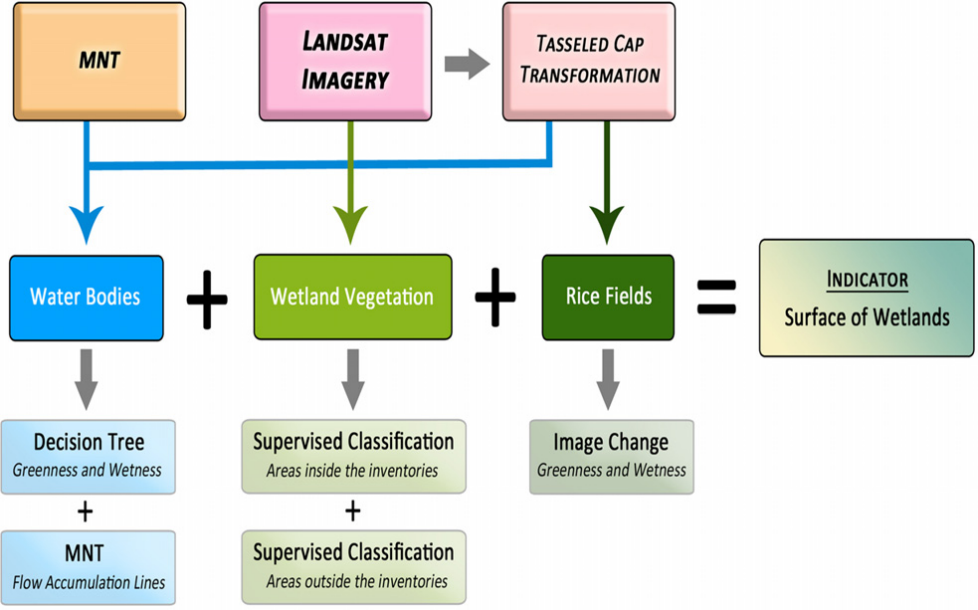
Diagram of the classification process developed for the wetland indicator.

Therefore, the indicator is basically the sum of three layers obtained with three different methods of classification (Fig 4).

Water bodies are detected using a decision tree based on the Greenness and Wetness indices of the TC transformation. Land and overlaying vegetation associated to water (wet areas) are detected through a supervised classification of Landsat imagery using the maximum likelihood algorithm. Rice fields are detected using a seasonal image change analysis based on vegetation dynamics provided by the Greenness and Wetness indices.

Each of these techniques is used with different parameters according to the properties of each image and the criteria of the researcher.

#### Binary masks

Binary masks are developed based on the spatial datasets defined earlier. These masks contain two types of information: presence of data, represented as 1, or absence of data, represented as 0. This selection is used to exclude areas that are not contributing to the analysis of the satellite reducing unnecessary processing and reducing the amount of errors. The following masks are used:

Inventory mask: a mask created for each department using the spatial information from the wetland inventories of the PACA region limiting the extension of certain analysis to the inventoried areas.

Slope mask: Based on the NMT data, a slope layer was developed presenting slopes lower or equal to 15%. The assumption was based on the presence of wetlands in areas presenting low slopes [14]. This layer is used in order to reduce errors in the classification process in mountainous regions within the PACA.

CLC masks: this mask was created by two supervised classifications of the Landsat imagery, one targeting the agricultural areas and another for urban areas. The extension of agriculture and urban areas of CLC 1990 and 2000 were used as a mask to cover the Landsat images. The resulting layers contain finer information on agriculture and urban areas as regions that CLC may overestimate the extension of these regions due to its coarse resolution or internal errors. Namely, in the case of the PNRC, CLC overestimates rice fields including areas that do not support this cultivation [15].

Due to their higher resolution (30 m), these new layers are a more accurate reference for agricultural and urban areas that are used to contrast the changes of wetlands. These layers also support in reducing some classifications errors when analyzing wetland areas, namely the errors in classifications between natural vegetation and crops or river beds and urban areas.

#### Water bodies: decision tree and flow accumulation lines

**Fig 5:**
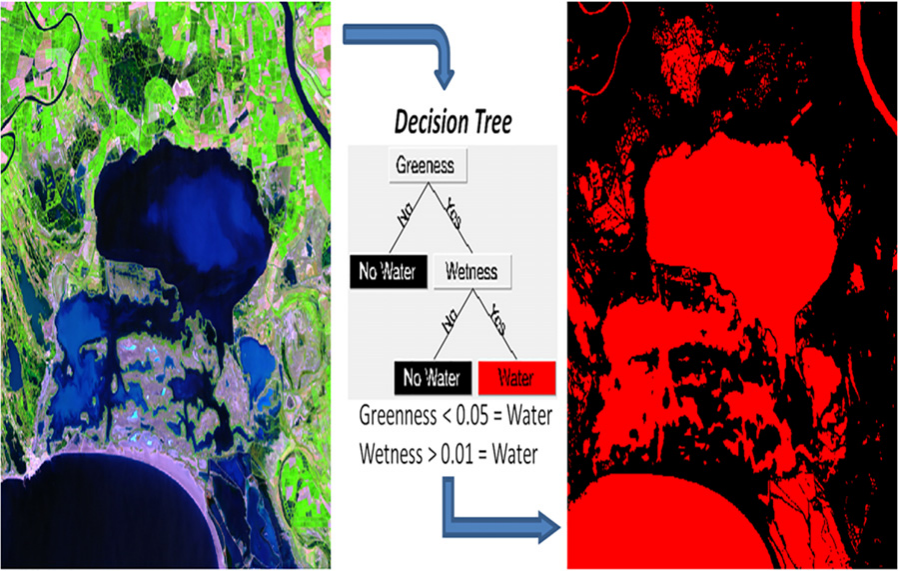
Decision tree generated for a Landasat 7 ETM+ image of the PNRC of July 2001. Red surface correspond to water bodies.

Greenness (G) and Wetness (W) are used in a decision tree (Fig 5) to distinguish between water areas, having a greenness less than zero and a wetness greater than zero (G < 0; W > 0), and no water areas, with greenness values higher than zero and wetness below zero (G > 0; W < 0). In order to avoid erroneous classifications, different thresholds are chosen based on the data of each satellite image as water presents different spectral responses according to the time of year (water depth, particles in suspension, etc.).

**Fig 6:**
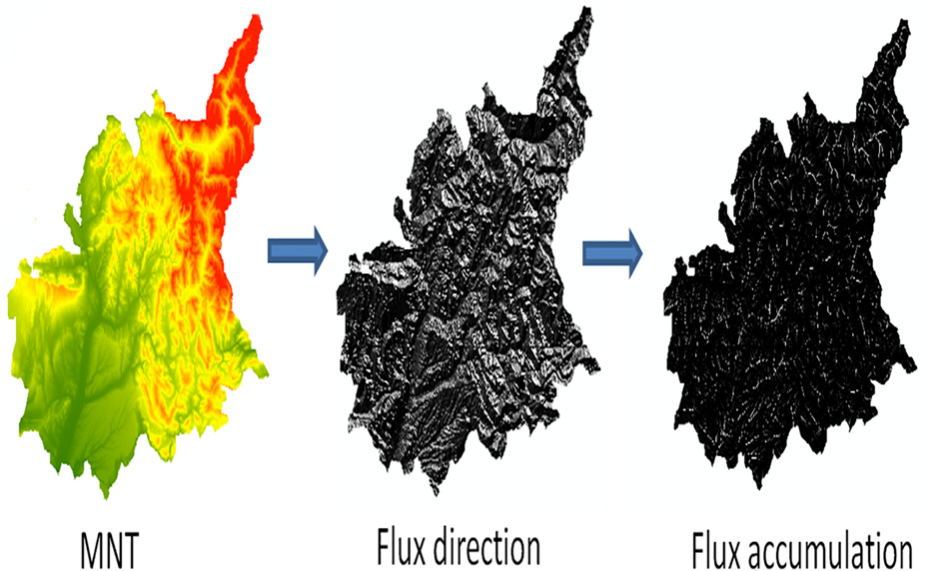
Calculation process of flux accumulation lines for Department 4.

The NMT is used to calculate the parameters of flow direction and flow accumulation lines. These parameters account the number of pixels that naturally drain into an outlet (pixel) marking the water flow. Once contrasted with the satellite information, this parameter proves to be a valid tool in identifying narrow linear features (channels and streams) that are not always detected by satellite images (Fig 6). An appropriate threshold is set, based on a visual analysis, for each image to reduce the amount of information reducing the error rates as there are a substantial number of small streams that may be omitted due to their low contribution to the major water flows. Thresholds range between 200–700 mm/year and the resulting information on water courses is added to the decision tree layer obtaining a full layer on water bodies.

#### Wetland vegetation: supervised classification

The supervised classification supports the process of separating soils and vegetation related to water bodies or wetlands from other land covers. The slope masks were used in the classification of the satellite images to minimize the effect of the mountains, as this process covers a larger number of land covers and is quite sensitive to the effects of the hill shades (i.e. shadows, bare rocks). Through this process we extracted different classes of wetland namely riparian forest, floodplains and river beds. This step includes two processes with different approaches depending on the analyzed area:

Areas inside the inventories are classified using the inventory masks, a supervised classification is performed only for the areas that are included in the wetlands inventories or in the wetland classes of the PNRC LULC layers. That way, it is possible to focus on representing wetlands. Lower thresholds can be used for the different classes making the classification more sensitive for wetland detection.

The second classification process is used to represent the areas outside the inventories (not inventoried as wetlands). In order to reduce the errors, the thresholds used for the classes are lower than the previous ones (less sensitive classification). The urban and agriculture mask derived from CLC was used in order to generate less classification confusions. Mask based on CLC 1990 was used for images from 1984 and mask of CLC 2000 for those from 2001.

#### Rice fields: image change

**Fig 7:**
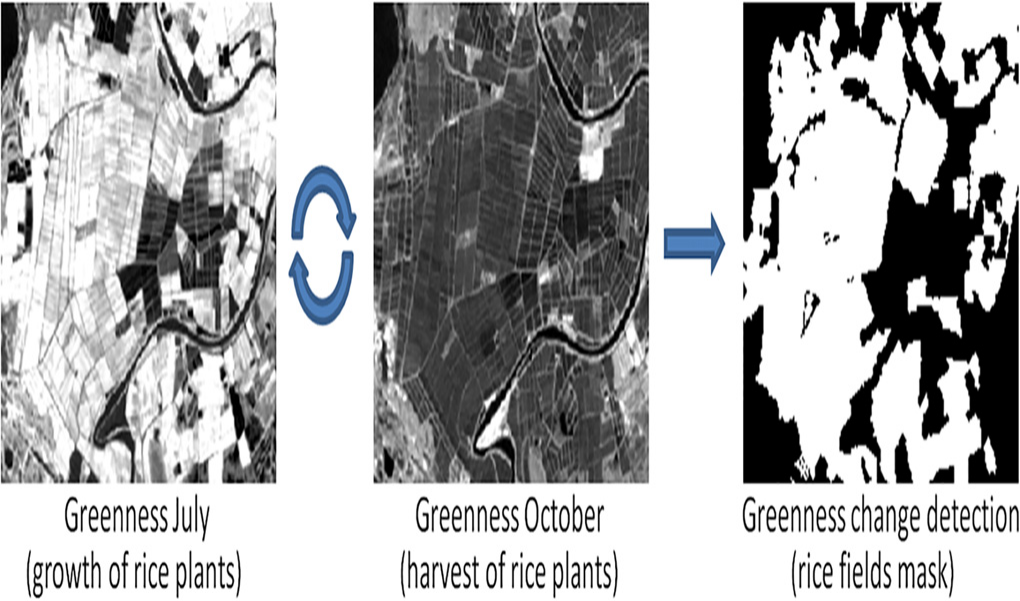
Image change process for rice fields detection in the PNRC between July and October, 2001. White areas correspond to a high change on greenness values representing rice fields.

Rice fields were analyzed comparing Greenness values between summer and autumn months. According to rice crops annual cycle [11], rice plants have their maximum height (growth) between July and August, while the harvest begins in late September. Therefore, the highest differences in this variable are observed during summer and early autumn periods. A change threshold is applied to differentiate and exclude other types of vegetation as they experience a much lower change in the G and W indices (Fig 7). The resulting layer represents the surface strictly covered by rice fields.

#### Surface of wetlands: union of layers

**Fig 8:**
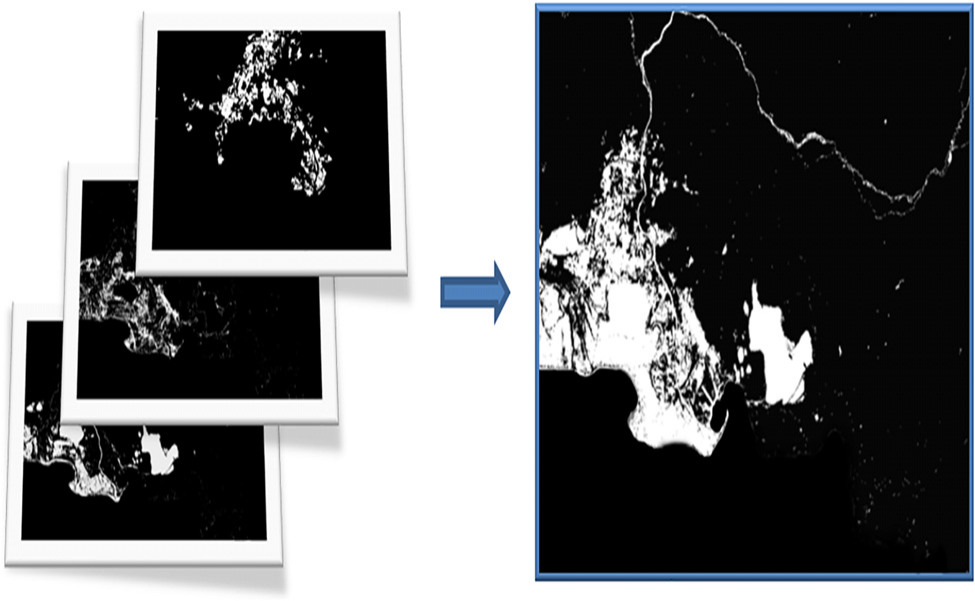
Example of the layer union process for Dep. 13. Water bodies, wetland vegetation and rice fields are combined to obtain the wetland indicator layer.

The resulting three layers on water bodies, wet areas and rice fields are combined to form the wetland indicator layer. The monthly images of each department are aggregated into one to get the total surface covered by wetlands in a complete annual cycle (Fig 8). The layer is the union of all the pixels detected as wetland by the indicator.

#### Evaluation of results

**Fig 9:**
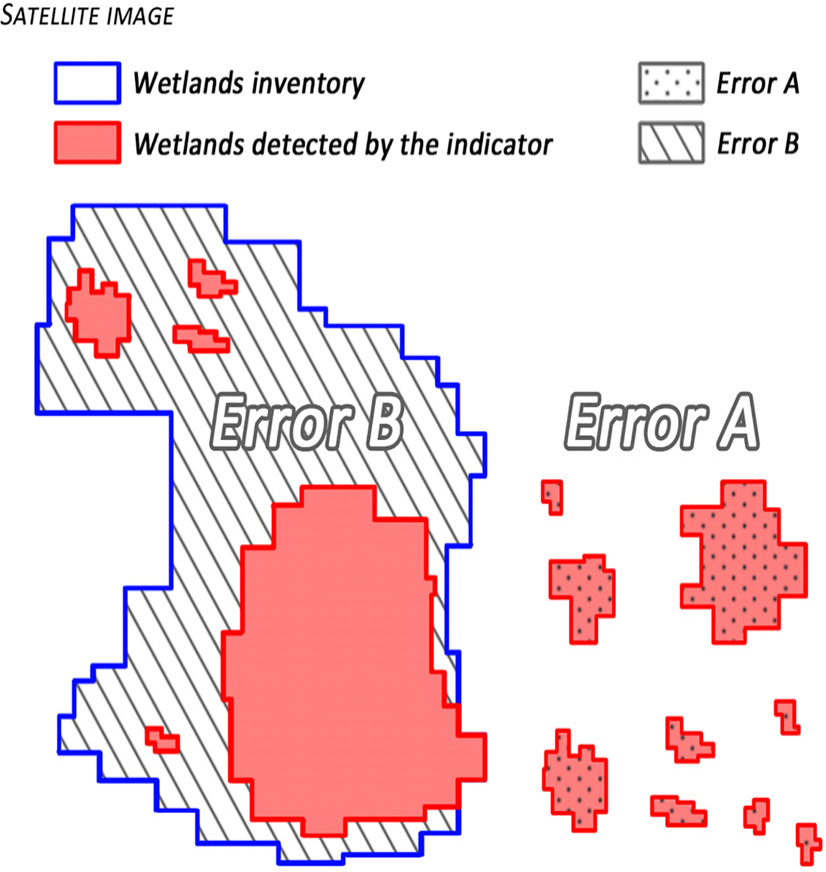
Illustrative example of error A and error B calculated from the layers of the indicator and the inventories of wetlands.

A ground truth process is applied to the final layer of the indicator. The results obtained for the years 1984 and 2001 are compared to the wetlands inventories in order to assess the reliability of the indicator. The classification results are validated using the available wetlands reference (inventories) where two types of errors (errors A and B) are calculated (Fig 9):

Error A is the percentage of land that is erroneously classified as wetland. This error is understood as the surface detected by the indicator that is outside the reference layers (not considered as wetland in the inventory). It would be the overestimation of wetlands compared with the inventories.

Error B corresponds to the percentage of real wetlands (present in the inventories) that are not detected by the indicator. In other words, this would be the surface of the references that is not classified as wetland (omission).

Due to the temporal gap between inventories (2000–2001) and images used for the indicator development, as comparisons are made, error A observed in 1984 may correspond to actual wetlands of that date that have been lost in the year 2000 so they are not included in inventories. Similarly, a portion of error B can be caused by new wetland areas generated between 1984 and 2001, and therefore they are not detected in the images of 1984.

## Results and Discussion

### Wetland indicator results

Wetland detection showed to exceed 80% of the inventoried surfaces in most of the studied departments for the years 1984 and 2001, although Dep. 84 showed a lower detection rate, around 70–76%.

**Fig 10:**
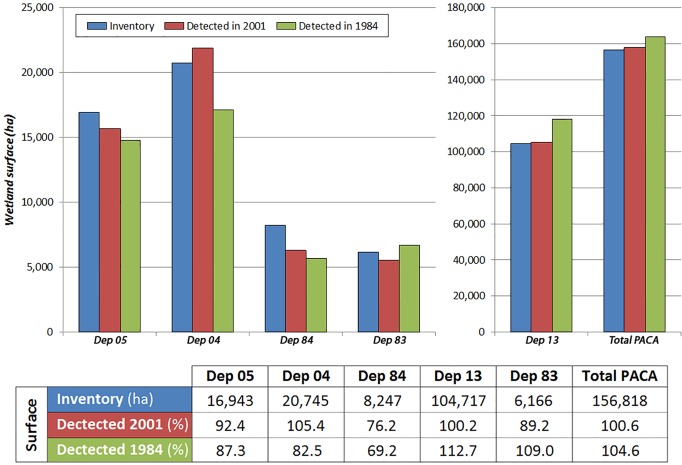
Indicator results in hectares. Years 1984 and 2001 are compared with the wetland inventories. Different scales are used in order to show in more detail the differences in surface detected by RS in each department.

Fig 10 shows the results of the level of detection of the indicator per department. Interannual differences exist, such as in the case of Dep. 04 or 83, where the detection varies by as much as 20%. Several reasons could explain this, such as changes in wetland areas (land use change), the quality of the images used in the classifications, or inaccuracies in the inventories. Considering the whole study area (the 5 departments.), the total wetland surface detected corresponds well with the inventories, with only a slight over-estimation: +4.6% in 1984, +0.6% in 2001.

**Fig 11:**
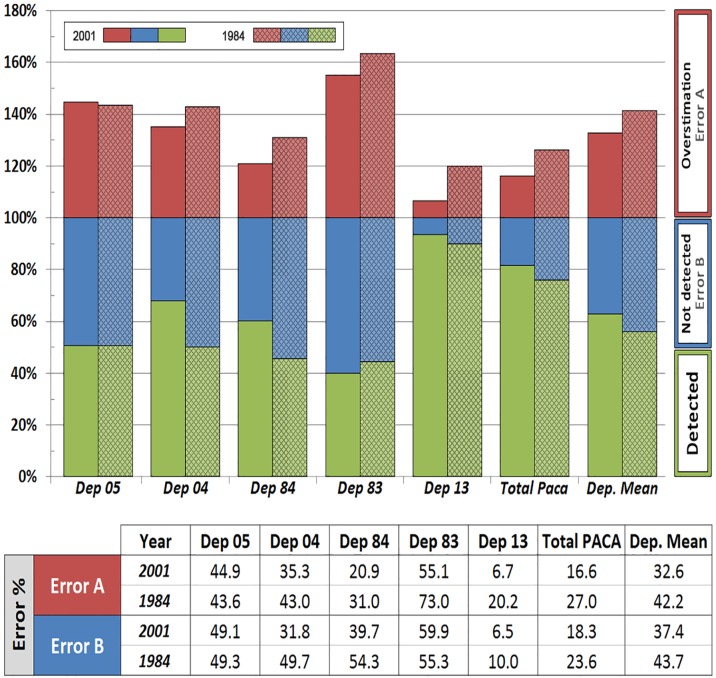
Results of Error A and B (%) obtained in the analysis of the wetland indicator in years 1984 and 2001.

Two types of errors have been calculated in the areas detected by the indicator. Fig 11 shows the percentage of errors varying between 6–73%. Dep. 13 shows the least percentage of errors, rounding 6% for both errors in year 2001; 20% for error A and 10% for error B in 1984. This is expected as remote sensing has proven to be a very efficient tool in areas with flat topography where the wetlands are large and well defined [16]. Wetlands in this area have good flood conditions throughout the year, being mainly large lagoons, marshes, salines and rice fields receiving the majority of the water contributions of the basin. Therefore, as results showed, it is an ideal area for wetland detection through RS techniques. The results in this department are really good, in line with, and improving in some cases, those obtained by other wetland indicators [2, 16, 17]. In fact it has achieved similar results to those achieved with SPOT-5 imagery in the PNRC region [18, 19]

Results for Dep. 13 contrast with those obtained for the alpine areas, especially in Dep. 05 and 04 whose error rates are much higher being around 30–50% for both errors A and B (success of 50–70%). These results are below the expected accuracy but are acceptable considering the topography of the area [20] but it does not reach the accuracy that other authors have succeeded in other areas of research [21, 22].

Dep. 84 shows big differences between error A and B, unlike the previous ones which were quite similar although slightly higher in error B, about 5%. Dep. 84 presents better numbers for overestimated areas (error A), 21% and 31% for years 2001 and 1984 respectively, being about 20% higher for B error, 40% in 2001 and 54% in 1984. Wetlands areas located in the Alpine region of the PACA are based mainly on rivers and streams, most of short length and width, with few areas of flooding. These are difficult to detect visually because of the resolution of the Landsat imagery. This would explain the higher percentage obtained for error B, since a large part of the surface inventoried in the field was not detected due to the limitations of the Landsat 30 m resolution imagery [23, 24]. Most wetlands that are easily detectable correspond to large water bodies, i.e. lakes or dams identified through the main channels. Satellite images of higher resolution have been shown to improve the accuracy of remote sensing studies on different topics [25, 26]. Therefore, it is expected that advances in the technology of satellites and the availability of higher quality images will bring improvements in the study of these departments.

Dep. 83 alto presents important percentages for errors A and B, being higher than 55%. Despite being a coastal department, it has a pronounced relief in the northern part presenting wet areas of small size which are difficult to detect. It is therefore another case limited by the topography and the small size of wetlands

Department 06 does not have an inventory of wetlands so far to perform the validation of the indicator. However, being a region very similar to Dep. 83 in terms of topography, probably error rates would be quite similar, being also the accuracy quite low compared to Dep. 13

The errors A and B calculated for the whole region (5 departments) are respectively 27% and 24% for the year 1984, and 17% and 18% for 2001 accounting for the differences in the spatial overlap between the inventories and the wetland indicator layers (Fig 9). Therefore, it is possible to say that the overall results are good and are in line with the studies consulted during this research.

As discussed above, the indicator shows more accurate results in large, well defined wetlands, especially in regions with minor slopes. Errors A & B range between 6–20% in Dept. 13, but between 20–73% in Depts. with complex topography, mainly due to shadow effects, the nature of the wetlands in these regions (i.e. mainly small or linear wetlands), and also the limitations on image resolution.

**Fig 12:**
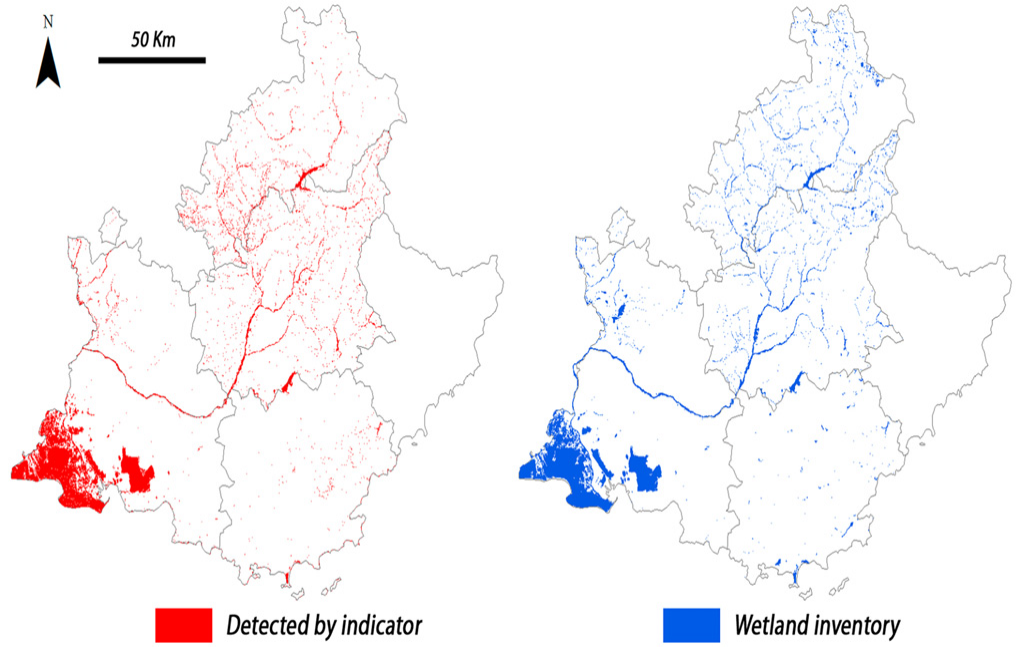
Map of wetlands of the PACA region. To the left, surface detected by the wetland indicator; to the right, inventory of wetlands.

Fig 12 compares wetlands mapped from inventories with the surface detected by the satellite-derived indicator. The maps show that the regional distribution of wetlands matches with reasonable accuracy from a visual standpoint; the satellite-based indicator seems to detect a higher amount of isolated wetlands in mountainous regions. But facing a regional mapping, the result can be considered quite acceptable.

It is also remarkable that despite the ranges of errors A (7%– 73%) and B (7%– 60%) (Fig 11) at department scale, the overall wetland surfaces detected at PACA scale in both years fit within 5% the surface inventoried (Fig 10). Both types of errors largely compensate each other, and when combined over a broad scale lead to an overall good approximation of wetland areas at the regional level.

Another positive aspect of the results is that the indicator reaches its highest accuracy in PRNC area where most wetlands are concentrated (67% of the PACA region) and where these have greater ecological, environmental and socioeconomic importance [27, 28]. The alpine areas (Dep. 05, 04 and 84) represent about 30% of the total wetland surface in the region, being a significant percentage. Given the nature of wetlands in the area, there are additional methods that could support the monitoring of wetlands such as streamflow and hydrometric monitoring systems [29].

### Evolution of wetlands between 1984 and 2001

Table 3 shows variations between 1984 and 2001 of surfaces and error rates obtained for the wetland indicator. Positive values correspond to increase in surface detected or error rates. Decreases are negative numbers, being in the case of error A and B improvements in the precision of detection.

**Table 3:** Variations in the wetland surface detected and in Error A and B between years 1984 and 2001.

	Dep 05	Dep 04	Dep 84	Dep 83	Dep 13	Total PACA	Dep. Mean
**Surf. Detected**	+5.1	+22.8	+7.0	-19.8	-12.5	-4.0	-
**Error A**	+1.3	-7.7	-10.1	-17.9	-13.5	-10.4	-9.6
**Error B**	-0.2	-17.9	-14.6	+4.6	-3.5	-5.3	-6.3

Positive values correspond to increases in error rates; negative numbers are improvements in the degree of detection.

The total surface of wetlands detected by the indicator for the PACA region declined by c. 4% between 1984 and 2001, resulting in a loss of 6,302 hectares of wetlands in the region. Error A variation between these dates is -10.4%, accounting for the loss of wetlands. These areas are not reflected in the inventories of the year 2000s and the indicator can support in providing an indication on where major reduction in wetland areas have occurred. Furthermore, this decline is not likely to be caused by the decrease in rice fields as the rice fields area has doubled overall in France between 1984 (9,400 ha) and 2001 (20,200 ha) (Centre Français du Riz, *pers*. *comm*.), with the Camargue accounting for the major part of the national total area.

At department level, the wetland surfaces detected within Dep. 04, 05 and 84 apparently increased, which is unlikely to have happened in reality given the overall pressure facing wetlands in SE France [11]. In Dep. 05, the increase is +5.1% whereas both error rates remain very similar. There is scientific evidence that classification products of Landsats 5 and 7 are very similar [30], so it is not something derived from the use of two different satellites. Therefore, these small variations probably are caused by differences in the classification parameters (thresholds), which depend on the researcher and visual characteristics of the image. The mountainous areas generate more classification errors, so it is logical to think there is a greater sensitivity on the thresholds and these could generate significant differences in results [17, 20]. Further research will be needed to better understand the effects that slight changes in the thresholds could have on the surface of wetland detected by the indicator.

In contrast, departments 04 and 84 present significant improvements in both error rates, but higher for Error B (i.e. less omission). This differential rate of improvement between Errors A and B may thus account for the increased wetland surface, through a better detection of existent wetlands.

An important aspect to consider is that we used the 2001 wetland mask to analyse the 1984 images, which means that when calculating the error A some wetland areas could be omitted. Therefore, a fraction of the error A obtained in 1984 is caused by true wetlands. This would explain a significant portion of the declines in the overestimation. Furthermore, results in both departments may be influenced by the classification parameters set by the researcher as in the case of Dep. 05.

In 2001, Dep. 13 shows a 12.5% decrease in the wetland surface detected compared to year 1984. Error A was reduced by 13.5%, but Error B only to a lesser extent (3.5%). In short, over-estimations were reduced significantly between 1984 and 2001, whereas omissions barely changed. So the apparent reduction of wetland surface between the 2 dates may be partly due to a methodological improvement (i.e. a reduction of the wetland overestimation, between 1984 and 2001), but possibly too to a true wetland loss.

In Dep. 83, apparent wetland losses were higher (–19.8%), the reduction in overestimations were higher still than in Dep. 13 (Error A: -17.9%) but the omissions (Error B) increased (+4.6%). Here again, the different evolution of Errors A/ B could therefore account for the apparent wetland loss—which could potentially include a real reduction component too.

**Fig 13:**
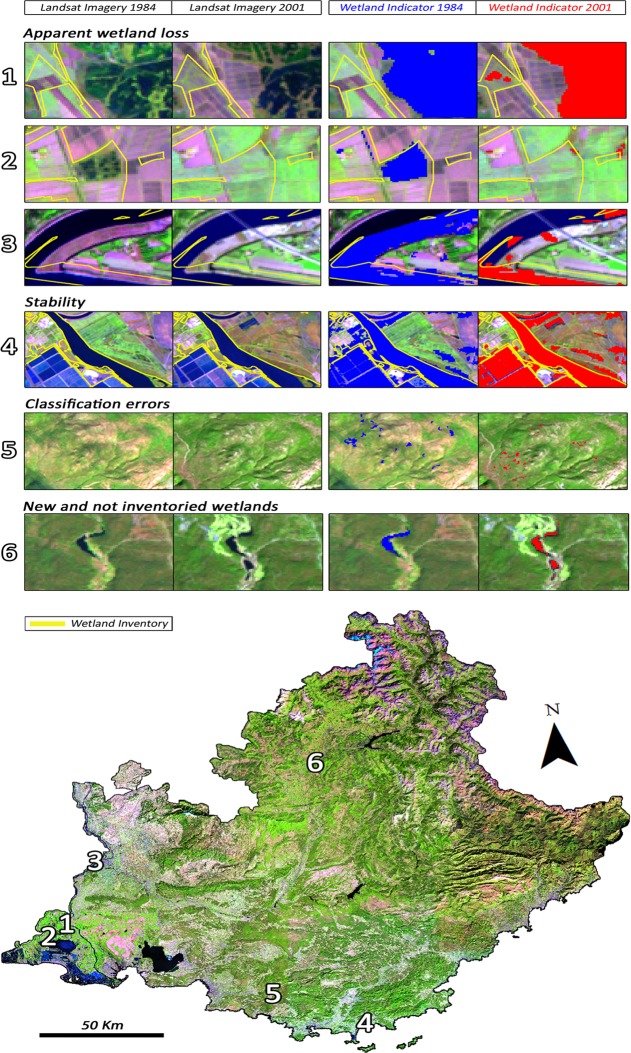
Cases of wetland classifications between 1984 and 2001.

Fig 13 shows specific cases of apparent wetland losses between the years 1984 and 2001 (images 1 to 3), cases of stability or no change (image 4), as well as overestimations caused by classification errors (images 5), and cases of wetlands detected by the indicators that were not inventoried (image 6) or generation of new ones (image 8).

The main limitation of the wetland indicator is related to the classification process, where confusions between wetlands and dry salinas and river banks might occur depending on the date of the satellite images used. Using NMT data helped overcome the limitations imposed by the topography (clouds, shadows, snow, and linear wetlands), but in the case of small wetlands, the indicator seems to reach its limits increasing the error of detection whenever the wetlands are smaller.

Another important methodological aspect is the use of wetlands inventories masks from 2001 and the absence of a previous inventory (80s or 90s), as this makes impossible an effective comparison of the results in both studied years as well as the correct analysis of changes in the surface of wetlands between 1984 and 2001.

For subsequent years (or previous) there will likely be no new wetland inventories available, only those used for this study. Wetlands outside the inventory should be detected by the satellite image as long as they have typical elements of a wetland that can be analysed (presence of water, vegetation changes, etc.). However, the surface detected will be corrupted by the real overestimation (classification errors). Therefore, this should be a limitation in the future as the method can identify wetland areas outside the current inventory but it is difficult to analyse how much of the error A is due to classification confusions or true wetlands. The layers obtained for areas outside the inventory should have a special treatment for a deeper analysis of the distribution of surface overestimated. On the other hand, if any area of the current inventory were no longer a wetland, it would not be detected in the new classifications (another land cover class would be detected instead).

## Conclusions

This research provides an overview of the benefits and limitations of one spatial indicator using satellite imagery and remote sensing techniques for identifying, inventorying and monitoring wetlands, as applied to a practical case study, i.e. wetlands of the PACA region.

Landsat imagery of 30 m resolution proved to support efficiently the identification of wetlands and therefore the calculation of total wetland area, although with different efficiency depending on areas. The application of this indicator with higher quality images could improve significantly the detection of wetlands in the region.

The approach works best for permanent wetlands in flat areas, but less so in areas with a complex topography, where shadows in mountainous areas and the presence of snow increase errors in the process of image classification. But despite this, at the regional scale, both the total surface detected (Fig 10) and the wetland spatial distribution (Fig 12) largely correspond with existing inventories, and demonstrate that a compensation exists between the two main types of errors studied. Therefore, the general results from our approach seem appropriate for estimating the surface area of wetlands at a regional scale, in an inventory perspective, and also have a similar accuracy to wetland indicators developed by other authors.

However, for monitoring purposes, i.e. detecting and interpreting wetland changes in time, further methodological improvements are needed. We were not yet able to separate unambiguously the potential impact of a change in error rates (A & B) from a real change in wetland surface area, e.g. in Depts. 13 and 83. Improving the protocol may require using less coarse field references for tests and validation, e.g. true field-checking. Here we used a single reference (i.e. a set of wetland inventories from the 2000’s) for ground-truthing our satellite-based results for 2 dates (1984 and 2001). This may be relatively valid for 2001, but less so for 1984, for which the “Errors” A & B may have been over- or underestimated, in case the wetland extent actually changed in the field between 1984 and the inventory date. Moreover, an inventory cannot be taken as representing with 100% accuracy the field reality in the year it is done, as some random field checks (not reported here) demonstrated.

This constitutes presently the main area to be further investigated before our approach can be applied for routine monitoring, together with the improvements required for regions with a marked topography and more linear, smaller wetlands, such as Alpine areas.

## References

[1] Taylor ARD, Howard GW, Begg GW. Developing wetland inventories in Southern Africa: A review. Vegetatio. 1995; 118: 57–79.

[2] Rebelo LM, Finlayson CM, Nagabhatla N. Remote sensing and GIS for wetland inventory, mapping and change analysis. Journal of environmental management. 2009; 90: 44–53. 10.1016/j.jenvman.2007.06.027 18367311

[3] Finlayson CM, Davidson NC, Spiers AG, Stevenson NJ. Global wetland inventory—status and future priorities. Marine and fresh water research. 1999; 5: 717–727.

[4] Lehner B, Doll P. Development and validation of a global database of lakes, reservoirs and wetlands. Journal of Hydrology. 2004; 296: 1–22.

[5] Davidson NC, Finlayson CM. Earth Observation for wetland inventory, assessment and monitoring. Aquatic Conservation: Marine and Freshwater Ecosystems. 2007; 17: 219–228.

[6] Ramsey E.W. Radar remote sensing of wetlands In: Lunetta RS, Elvidge CD editors. Remote sensing change detection: environmental monitoring methods and applications. Ann Arbor Press, Michigan, US; 1998 pp 211–243.

[7] MacKay H, Finlayson CM, Fernández-Prieto D, Davidson N, Pritchard D, Rebelo LM. The role of Earth Observation (EO) technologies in supporting implementation of the Ramsar Convention on Wetlands. Journal of environmental management. 2009; 90: 2234–42. 10.1016/j.jenvman.2008.01.019 18462862

[8] Xie Z, Xu X, Yan L. Analyzing qualitative and quantitative changes in coastal wetland associated to the effects of natural and anthropogenic factors in a part of Tianjin, China. Estuarine, Coastal and Shelf Science. 2010; 86: 379–386.

[9] Toyra J, Pietroniro A. Towards operational monitoring of a northern wetland using geomatics-based techniques. Remote Sensing of Environment. 2005; 97: 174–191.

[10] Ju J, Roy DP. The availability of cloud-free Landsat ETM+ data over the conterminous United States and globally. Remote Sensing of Environment. 2008; Vol. 112(3): 1196–1211.

[11] Tourenq C, Bennetts RE, Kowalski H, Vialet E, Lucchesi JL, Kayser Y, et al Are ricefields a good alternative to natural marshes for waterbird communites in the Camargue, southern France?. Biological Conservation. 2001; 100: 335–343.

[12] Kauth RJ, Thomas GS. The tasseled Cap—A Graphic Description of the Spectral-Temporal Development of Agricultural Crops as Seen by LANDSAT Proceedings of the Symposium on Machine Processing of Remotely Sensed Data. Purdue University of West Lafayette, Indiana; 1976 pp. 4B41–4B51.

[13] Giles PT. Remote sensing and cast shadows in mountainous terrain. Photogrammetric engineering and remote sensing. 2001; 7: 833–839.

[14] Rodhe A, Seibert J. Wetland occurrence in relation to topography: a test of topographic indices as moisture indicators. Agricultural and Forest Meteorology. 1999; 98–99: 325–340.

[15] Perennou C, Beltrame C, Guelmami A, Tomas Vives P, Caessteker P. Existing areas and past changes of wetland extent in the Mediterranean Region: and overview. Ecologia Mediterranea. 2012; Vol. 38(2): 53–66.

[16] Sl Ozesmi, Bauer ME. Satellite remote sensing of wetlands. Wetlands Ecology and Management. 2002; 10: 381–402.

[17] Zhang Y, Lu D, Yang B, Sun C, Sun M. Coastal wetland vegetation classification with a Landsat Thematic Mapper image. International Journal of Remote Sensing. 2011; Vol. 32(2): 545–561. 10.1080/01431160903475241

[18] Davranche A, Poulin B, Lefebvre G. Mapping flooding regimes in Camargue wetlands using seasonal multispectral data. Remote Sensing of Environment. 2013; Vol. 138: 165–171. 10.1016/j.rse.2013.07.015

[19] Davranche A, Lefebvre G, Poulin B. Wetland monitoring using classification trees and SPOT-5 seasonal time series. Remote Sensing of Environment. 2010; Vol. 114(3): 552–562. 10.1016/j.rse.2009.10.009

[20] Weiss DJ, Walsh SJ. Remote Sensing of Mountain Environments. Geography Compass. 2009; Vol. 3(1): 1–21.

[21] Wimmer A, Schardt M, Ziegler M, Ruppert G, Granica K, Schmitt U et al International Archives of Photogrammetry and Remote Sensing. 2000; Vol. 33, B7.

[22] Liu YS, Hu YC, Peng LY. Accurate Quantification of Grassland Cover Density in an Alpine Meadow Soil Based on Remote Sensing and GPS. Pedosphere. 2005; 15(6): 778–783.

[23] Wijedasa LS, Sloan S, Michelakis DG, Clements GR. Overcoming Limitations with Landsat Imagery for Mapping of Peat Swamp Forests in Sundaland. Remote Sensing. 2012; 4: 2595–2618. 10.3390/rs4092595

[24] Avitabilea V, Baccinib A, Friedlc MA, Schmulliusa C. Capabilities and limitations of Landsat and land cover data for aboveground woody biomass estimation of Uganda. Remote Sensing of Environment. 2012; Vol. 117: 366–380. 10.1016/j.rse.2011.10.012

[25] Camathias L, Bergamini A, Küchler M, Stofer S, Baltensweiler A. High-resolution remote sensing data improves models of species richness. Applied Vegetation Science. 2013; Vol. 16(4): 539–551. 10.1111/avsc.12028

[26] Mahavir D. High (spatial) resolution vs. Low resolution images: a planner’s view point. International Archives of Photogrammetry and Remote Sensing. 2000; Vol. 33, B7.

[27] Aznar JC, Dervieux A, Grillas P. Association between aquatic vegetation and landscape indicators of human pressure. Wetlands. 2003; Vol. 23(1): 149–160. 10.1672/0277-5212(2003)023

[28] Dervieux A. La difficile gestion globale de l'eau en Camargue (France): le Contrat de delta. Vertigo. 2005; Vol. 6(3). 10.4000/vertigo.2411

[29] Halverson M, Fleming S. Complex networks, streamflow, and hydrometric monitoring system design. Hydrology and Earth System Sciences. 2014; 11: 13663–13710. doi: 10.5194/hessd-11-13663-2014, 2014

[30] Vogelmann JE, Helder D, Morfitt R, Choate MJ, Merchant JW, Bulley H. Effects of Landsat 5 Thematic Mapper and Landsat 7 Enhanced Thematic Mapper Plus radiometric and geometric calibrations and corrections on landscape characterization. Remote Sensing of Environment. 2001; 78: 55–70.

